# A combined vaccine approach against *Vibrio cholerae* and ETEC based on outer membrane vesicles

**DOI:** 10.3389/fmicb.2015.00823

**Published:** 2015-08-11

**Authors:** Deborah R. Leitner, Sabine Lichtenegger, Philipp Temel, Franz G. Zingl, Desiree Ratzberger, Sandro Roier, Kristina Schild-Prüfert, Sandra Feichter, Joachim Reidl, Stefan Schild

**Affiliations:** Institute of Molecular Biosciences, University of GrazGraz, Austria

**Keywords:** outer membrane vesicles, *Vibrio cholerae*, enterotoxigenic *Escherichia coli*, vaccines, enteric pathogens, combined vaccine

## Abstract

Enteric infections induced by pathogens like *Vibrio cholerae* and enterotoxigenic *Escherichia coli* (ETEC) remain a massive burden in developing countries with increasing morbidity and mortality rates. Previously, we showed that the immunization with genetically detoxified outer membrane vesicles (OMVs) derived from *V. cholerae* elicits a protective immune response based on the generation of O antigen antibodies, which effectively block the motility by binding to the sheathed flagellum. In this study, we investigated the potential of lipopolysaccharide (LPS)-modified and toxin negative OMVs isolated from *V. cholerae* and ETEC as a combined OMV vaccine candidate. Our results indicate that the immunization with *V. cholerae* or ETEC OMVs induced a species-specific immune response, whereas the combination of both OMV species resulted in a high-titer, protective immune response against both pathogens. Interestingly, the immunization with *V. cholerae* OMVs alone resulted in a so far uncharacterized and cholera toxin B-subunit (CTB) independent protection mechanism against an ETEC colonization. Furthermore, we investigated the potential use of *V. cholerae* OMVs as delivery vehicles for the heterologously expression of the ETEC surface antigens, CFA/I, and FliC. Although we induced a detectable immune response against both heterologously expressed antigens, none of these approaches resulted in an improved protection compared to a simple combination of *V. cholerae* and ETEC OMVs. Finally, we expanded the current protection model from *V. cholerae* to ETEC by demonstrating that the inhibition of motility via anti-FliC antibodies represents a relevant protection mechanism of an OMV-based ETEC vaccine candidate *in vivo*.

## Introduction

Enteric infections associated with diarrhea remain a leading global health problem. They have been recognized as the first leading cause of neonatal diarrhea and the second major cause of infant mortality in developing countries (Rao et al., 2003; Bryce et al., 2005; Levine, 2006). Besides, diarrheal disease also represents the most common health problem in travelers to low resource countries. Almost half of all diarrheal infections are caused by the enterotoxin producing Gram-negative bacteria *Vibrio cholerae* or enterotoxigenic *Escherichia coli* (ETEC) (Sánchez and Holmgren, 2005; WHO, 2006). In general the pathophysiology of both pathogens is quite similar. They enter the human host via contaminated food or water, passage through the stomach and finally reach the intestinal tract representing the primary colonization site. Both pathogens attach to the mucosal surface via surface-associated structures, i.e., the toxin-coregulated pilus in case of *V. cholerae* or various fimbrial colonization factors (CFs) in case of ETEC. The secretory diarrhea is mainly caused by closely related AB-toxins known as cholera toxin (CT) or heat-labile enterotoxin (LT) for *V. cholerae* or ETEC, respectively (Faruque et al., 1998; Sánchez and Holmgren, 2005; Fleckenstein et al., 2014).

*V. cholerae* is the causative agent of the severe secretory diarrheal disease cholera, which can lead to death within hours, if left untreated (Koch, 1884). Although oral and intravenous rehydration therapies are effective, the access to these treatments is limited in regions with poor infrastructure or in the course of rapidly spreading outbreaks (Harris et al., 2012). Thus, the WHO estimates 3–5 million cholera cases and 100,000–120,000 cholera deaths every year (WHO, 2010). While cholera might be the more severe disease, infections with ETEC are responsible for the largest number of diarrheal episodes. Conservative estimates suggest that ETEC strains are incriminated as the cause of 280–400 million diarrheal cases, resulting in 300,000–500,000 deaths every year mostly in children under the age of 5 years. ETEC is also the major cause of travelers' diarrhea, being responsible for one-third to one-half of all diarrheal episodes in travelers to Africa, Asia, and Latin America, including both civilian and military personnel (WHO, 2006). These epidemiological numbers highlight the desperate need for a cheap and effective vaccine against both pathogens.

Currently, there are two types of oral cholera vaccines available (WHO, 2010). Dukoral is most widely used and consists of heat- or formalin-killed whole cells of *V. cholerae* serogroup O1 and a recombinant produced CTB. Through its CTB component, it represents the only available vaccine, which affords short-term cross-protection against LT producing ETEC strains (Clemens et al., 1988, 1990; Peltola et al., 1991; Jertborn et al., 1992, 1993, 1994). However, the short shelf life, high costs, and its need for cold-chain distribution restrict the vaccine for broad use in developing countries (Cumberland, 2009; Bishop and Camilli, 2011). Shanchol and mORCVAX are closely related and use identical strains but are produced by independent manufacturers and diverging methods. These vaccines are thought to confer longer term protection, but still require a cold-chain. In addition, they do not contain the CTB subunit and will therefore not protect against ETEC (WHO, 2010). In contrast, there is no licensed vaccine available against ETEC (Svennerholm and Lundgren, 2012). Several ETEC vaccine strategies have focused on a relatively small group of antigens, namely, plasmid encoded surface antigens known as CFs or LT. Along with attempts to develop LT-based toxoids, oral live attenuated, or inactivated whole cell vaccines overexpressing the most prevalent CFs have been the central focus of the vaccine development and are currently in different clinical trials (Tobias et al., 2010a,b, 2011; Harro et al., 2011; Turner et al., 2011; Darsley et al., 2012; Byrd and Boedeker, 2013; Holmgren et al., 2013; Lundgren et al., 2013).

Recently, we introduced and characterized outer membrane vesicles (OMVs) derived from *V. cholerae* as an alternative vaccine candidate against cholera (Schild et al., 2008, 2009; Bishop et al., 2010; Leitner et al., 2013). OMVs are naturally released by various Gram-negative bacteria and largely reflect the composition of the outer membrane (OM) and the periplasm of their donor bacterium (Gankema et al., 1980; Beveridge, 1999; Ellis and Kuehn, 2010). Thus, they represent a combination of various native surface antigens, which are highly immunogenic as well as self-adjuvant. For these reasons, OMVs have been studied for their immunogenic and protective properties of several bacterial species. In case of *V. cholerae* OMVs, the induction of a specific, high-titer protective immune response against the respective pathogen upon intranasal immunization of mice has been successfully demonstrated (Schild et al., 2008, 2009). Further studies characterized the O antigen to be the dominant protective antigen and revealed a protection mechanism based on inhibition of motility by anti-O antigen antibodies binding to the sheathed flagellum of *V. cholerae* (Bishop et al., 2010; Leitner et al., 2013). Interestingly, a recent study by Roy et al. indicated that immunization with ETEC OMVs derived from a flagellar mutant also induced a robust immune response in the murine model (Roy et al., 2011).

Intrigued by this report, the present study aimed to create an improved, combined vaccine against the two major bacterial infectious agents of diarrheal diseases: *V. cholerae* and ETEC. Our results indicate, that immunization with a mixture of detoxified and enterotoxin-negative OMVs derived from *V. cholerae* and ETEC induces a high-titer, protective immune response against both pathogens. Interestingly, *V. cholerae* OMVs alone already induce a so far uncharacterized and CTB independent protection against ETEC colonization. Additionally, we used *V. cholerae* OMVs as antigen delivery vehicles for the heterologously expressed ETEC surface antigens CFA/I and FliC. Although a detectable immune response against both heterologously expressed ETEC antigens was induced, none of these approaches resulted in an improved protection compared to a simple *V. cholerae* and ETEC OMV mixture. Finally our data indicates that inhibition of motility based on anti-FliC antibodies might also play a role in the protection mechanism against ETEC.

## Materials and methods

### Bacterial strains, plasmids, and growth conditions

Bacterial strains and plasmids used in this study are listed in Table 1; oligonucleotides are listed in Table 2. *V. cholerae*, AC53, a spontaneous streptomycin (Sm)-resistant mutant of the clinical isolate E7946 (O1 El Tor Ogawa), or ETEC H10407-S, a Sm-resistant mutant of the clinical isolate H10407, were used as wild type strains. *E. coli* strain DH5αλ*pir* and SM10λ*pir* were used for genetic manipulations. If not noted otherwise, strains were cultured in Luria Bertani (LB) broth or on LB broth agar plates with aeration at 37°C. For optimal induction of the enterotoxins, *V. cholerae* was grown in AKI broth and ETEC was grown in CYE-G media [casamino acids (30 g/l), yeast extract (3 g/l), K_2_HPO_4_ (0.5 g/l), glucose (2 g/l) (pH 8), (Sack et al., 1980; Iwanaga and Yamamoto, 1985; Iwanaga et al., 1986; Leitner et al., 2013)]. To induce CFA/I expression, ETEC was cultivated on CFA plates (1% casamino acids, 0.15% yeast extract, 0.005% MgSO_4_, 0.0005% MnCl_2_, 1.5% agar) (Evans et al., 1977; Horstman and Kuehn, 2000; Baker et al., 2009). If required, antibiotics, or other supplements were used in the following final concentrations: Sm, 100 μg/ml; Ampicillin (Ap), 100 μg/ml or in combination with other antibiotics 50 μg/ml; kanamycin (Km), 50 μg/ml; chloramphenicol (Cm), 30 μg/ml; IPTG, 1 mM; arabinose (Ara), 0.02%; glucose (Gluc), 0.2%; sucrose (Suc), 10%.

**Table 1:** **Strains and plasmids used in this study**.

**Strain or plasmid**	**Genotype/resistance/description**	**References**
***E. COLI***		
DH5αλpir	F^−^Φ80Δ*lacZ*Δ*M15*Δ(*argF lac*)*U169 deoR recA1 endA1 hsdR17* (r^−^_K_m^+^_K_) *supE44 thi-1 gyrA69 relA1*, λ*pir*R6K, Ap^r^	Hanahan, 1983
SM10λpir	thi thr leu tonA lacY supE recA::RPA-2-Te::Mu λpirR6K, Km^r^	Miller and Mekalanos, 1988
EWT	H10407-S, wild type ETEC strain; serotype O78:H11; CFA/I LT^+^ STh^+^ STp^+^, Sm^r^	Evans and Evans, 1973
EΔ*msbB*	H10407 Δ*msbB*; Sm^r^	This study
EΔ*msbB*Δ*eltA*	H10407 Δ*msbB*Δ*eltAB* eltA::pGP704; Sm^r^, Ap^r^	This study
***V. CHOLERAE***		
VWT	AC53, wild type *V. cholerae* strain serogroup, O1; biotype, El Tor; serotype, Ogawa; spontaneous Sm^r^ mutant of E7946; clinical isolate from Bahrain 1978; *hapR*^+^, Sm^r^; used for previous immunization studies (Schild et al., 2008, 2009; Bishop et al., 2010, 2012; Leitner et al., 2013)	Miller et al., 1989
VΔ*msbB*Δ*ctxAB*	AC53 Δ*msbB*Δ*ctxAB*; Sm^r^	This study
VΔ*msbB*Δ*ctxAB*Δ*flaA*	AC53 Δ*msbB*Δ*ctxAB*Δ*flaA*; Sm^r^	This study
**PLASMIDS**		
pCVD442	ori6K mobRP4 sacB, Ap^r^	Donnenberg and Kaper, 1991
pGP704	ori6K mobRP4, Ap^r^	Miller and Mekalanos, 1988
pΔctxAB	pCVD442::ΔRS1-CTX phage-TLC of AC53, Ap^r^	Bishop et al., 2010
pΔmsbB_E	pCVD442::ΔmsbB of H10407, Ap^r^	This study
pΔeltAB	pCVD442::ΔeltAB of H10407, Ap^r^	This study
eltA::pGP704	Insertion of pGP704 in *eltA* of H10407	This study
pΔflaA	pCVD442::ΔflaA of AC53, Ap^r^	This study
pCFA/I	CFA/I of H10407 cloned into pBAD18, Km^r^	This study
pCfaB	CfaB of H10407 cloned into pQE30, Ap^r^	This study
pFliC	1320-bp *fliC* fragment cloned into pQE30, Ap^r^	This study
pFlaA-FliC	Hybrid construct consisting of FlaA (first 47 aa) of AC53 and FliC (last 440 aa) of H10407 cloned into pBAD18, Km^r^	This study
pAR181	gfpmut3b^*^ gene under transcriptional control of the *E. coli* rrnB-P1 promoter	Sherlock et al., 2004

**Table 2:** **Oligonucleotides used in this study**.

**Primer Name**	**Sequence (5^′^–3^′^)^a^**
msbB_E_SacI_1	TTGAGCTCTTAACCAGCGCCGAAGTG
msbB_E_BamHI_2	TAGGATCCCATGCTTTTCCAGTTTCGGAT
msbB_E_BamHI_3	TTGGATCCTAAAAAAGCCTCTCGCGA
msbB_E_XbaI_4	TTTCTAGAGCTGTGATGCTGTCTGC
eltAB_SacI_1	TTGAGCTCTCCGGCAGGGTGAAGAC
eltAB_BamHI_2	TTGGATCCCATCGAGGAAAACTTATATAT
eltAB_BamHI_3	TTGGATCCGAAAACTAGTTTGCTTTAAAAG
eltAB_XbaI_4	TTTCTAGACATCATCGTTGGTCGTGAC
eltA_XbaI_1	ATTCTAGACCCCCAGATGAAAT
eltA_SacI_2	TTGAGCTCGCCTCTTAACTTTTGATTGATA
flaA_SacI_1	TTGAGCTCAAATGGCTATCAGTTAGAGC
flaA_BamHI_2	TTGGATCCCATAGTTTGCTCTCCTATCG
flaA_BamHI_3	TTGGATCCTAGTTCACGGTACCTTCATT
flaA_XbaI_4	TATCTAGATGAATCGCAACACGGTCAG
CFAI_StuI_1	ATAGGCCTTGATTTATTATTGATGGAAGCTCA
CFAI_NheI_2	ATGCTAGCTCTAGAGTGTTTGACTACTTGG
CfaB_SphI_fw	ATGCATGCGTAGAGAAAAATATTACTGTAGC
CfaB_HindIII_rv	TATAAGCTTAAGAATCAGGATCCCAAAGT
Flic_BamHI_fw	TAGGATCCCAGGCGATTGCTAACCGTT
Flic_SalI_rv	TAGTCGACACGCAGCAGAGACAGTACG
FlaA_FliC_SacI_1	TTTGAGCTCGAACTCGATAGGAGAGCAA
FlaA_FliC_2	AACGGTTAGCAATCGCCTGGCCTGCCGCGTCATCTTT
FlaA_FliC_3	AAAGATGACGCGGCAGGCCAGGCGATTGCTAACCGTT
FlaA_FliC_XbaI_4	TAATCTAGAGGTTGAGCGATAAGTGTAAA
qRT-PCR^b^	
Il6-fw	GAGGATACCACTCCCAACAGACC
Il6-rv	AAGTGCATCATCGTTGTTCATACA
Tnf-alpha-fw	CATCTTCTCAAAATTCGAGTGACAA
Tnf-alpha-rv	TGGGAGTAGACAAGGTACAACCC
Il1b(M)-fw	CAACCAACAAGTGATATTCTCCATG
Il1b(M)-rv	GATCCACACTCTCCAGCTGCA
36B4-fw	GCTTCATTGTGGGAGCAGACA
36B4-rv	CATGGTGTTCTTGCCCATCAG

a *Restriction sites are underlined*.

b *According to Leitner et al. (2013)*.

### Construction of deletion mutants and expression plasmids

Genetic manipulations including the isolation of chromosomal, plasmid, or PCR product DNA, PCR assays, the construction of in-frame deletion mutants, insertion mutants, and expression plasmids were carried out as described previously using derivatives of pCVD442, pGP704, or pBAD18 (Fengler et al., 2012; Leitner et al., 2013). The expression plasmid pFliC was constructed using the oligonucleotides flic_BamHI_fw and flic_SalI_rev for amplifying the respective gene, digested with BamHI and SalI, and ligated into pQE30, which has been digested with the same enzymes. The fusion protein FlaA-FliC was generated by SOE PCR (splicing by overlap extension), using chromosomal DNA of AC53 and H10407-S as a template and the oligonucleotide pairs flaAfliC_SacI_1 and flaAflic_2 as well as flaAfliC_3 and flaAfliC_XbaI_4 (Horton et al., 1989). The generation of overlapping regions allowed the annealing of the two PCR fragments in a further PCR reaction. The resulting PCR product was ligated into the SacI/XbaI-digested pBAD18. Introducing the GFP expression plasmid, pAR181, into the wild type strain of ETEC, generated a GFP expression strain.

### Preparation of OMVs, whole cell lysates (WCL), and LPS

*V. cholerae* as well as ETEC OMVs were isolated as previously described (Schild et al., 2008; Leitner et al., 2013). WCL were prepared as previously published using 0.1 mm glass beads in combination with a PowerLyzer™ 24 (MO BIO Laboratories, Inc.), applying three 1 min cycles at 3400 rpm with 1 min intervals on ice between each cycle (Roier et al., 2012, 2013). The protein concentrations of the OMV preparations were determined by measuring the absorbance at 260 and 280 nm using a Beckman Coulter DU730 spectrophotometer in combination with a TrayCell (Hellma) and the Warburg-Christian equation. The purification of the LPS of *V. cholerae* as well as ETEC cultures and the estimation of their concentrations were performed as previously reported (Leitner et al., 2013).

### Purification of proteins and immunization

The His-tagged proteins were purified using the expression plasmid pCfaB and pFliC as described previously (Leitner et al., 2013). In order to generate high-titer IgG1 antisera against CfaB, the purified His-tagged protein (100 μg protein per immunization) was used to intraperitoneally immunize mice on days 0, 14, and 28. Finally, sera were collected on day 40.

### Trichloroacetic acid precipitation (TCA)

To precipitate the CT as well as the LT, cultures were grown under toxin expressing conditions and centrifuged (6500 × *g*, 25 min, 4°C). The toxin-containing supernatant was collected, filtered and mixed with a 100% TCA-solution (final concentration 20%). After centrifugation (22,500 × *g*, 60 min, 4°C), the pellet was washed twice with acetone, air-dried, dissolved in PBS (pH 7.4) and stored at −20°C.

### TNF-**α**, IL-1ß, and IL-6 induction assay

The stimulation of RAW macrophages with ETEC OMVs derived either from WT or Δ*msbB* (1 μg/ml), the cDNA synthesis as well as the qRT-PCR were performed as described previously (Leitner et al., 2013).

### Animals

Female BALB/c mice (Charles River Laboratories) were used in all experiments in accordance with the rules of the ethic committee at the University of Graz and the corresponding animal protocol, which has been approved by the Austrian Federal Ministry of Science and Research Ref. II/10b. Mice were housed with food and water *ad libitum* and monitored under the care of full-time staff. All animals were acclimated for 1 week before any procedures were carried out and were approximately 9 weeks old at the start of the immunization.

### OMV immunization protocol and neonatal challenge with *V. cholerae* and ETEC

Nine-week-old female mice were intranasally immunized with either one OMV type (25 μg) or with an OMV mixture (12.5 μg each) in PBS at days 0, 14, and 28 as described previously (Schild et al., 2008; Leitner et al., 2013). A group of sham (PBS)-immunized control mice were housed in parallel with the immunized mice for the duration of each experiment. Mice were anesthetized by inhalation of isoflurane gas (2.5%) prior to all immunizations. Overall two independent immunization rounds for each immunization group were performed with at least three mice per group. Comparison of the results from the independent immunization rounds revealed no differences in the induction of a humoral immune response or the reduction of the colonization level in the respective immunization group.

Non-vaccinated control and immunized mice were mated at day 41, and 5- to 6-day-old neonates were challenged either with *V. cholerae* or ETEC according to a previously published method with slight modifications (Leitner et al., 2013). This indirect protection assay was used, since adult mice are only successfully colonized by *V. cholerae* after pretreatment with antibiotics to decrease the bacterial gut flora. For the preparation of the ETEC inoculum, bacteria were streaked on an LB-Sm plate (2 days prior to the infection), transferred to a CFA-Sm plate (16 h prior to the infection), diluted to the appropriate density in LB broth, and plated to determine the number of input CFU. In order to gain challenge data for both organisms, neonates from a given litter were splitted and orally infected with either *V. cholerae* or ETEC. The exact input doses ranged from 6.8 × 10^4^ to 1.6 × 10^5^ CFU/mouse for *V. cholerae* or 5.7 × 10^4^ to 1.5 × 10^5^ CFU/mouse for ETEC, which is in both cases at least 100-fold above the 50% infective dose (ID_50_) (Goldhar et al., 1986; Schild et al., 2008). Infected mice were given back to their respective dams. At 24 h post-infection, neonates were sacrificed by cervical dislocation, and the small intestine was removed from each neonatal mouse by dissection and was mechanically homogenized in 1 ml LB with 20% glycerol. Appropriate dilutions were plated on LB-Sm plates to determine viable counts. The limit of detection was 10 CFU/small intestine.

Additionally, a passive protection study using purified antisera in combination with the infant mouse model was performed with slight modifications as previously described (Leitner et al., 2013). The anti-IgG1 titer of each serum was determined via ELISA using the appropriate coating antigen (OMVs, His-Flic) to allow an adjustment to equal concentration in the assay. For the inoculum, an ETEC culture was adjusted to an OD_600_ of 0.003 and subsequently mixed in a 2:1 ratio with appropriate dilutions of non-vaccinated control, anti-VΔ*msbB*Δ*ctxAB* OMV, anti-EΔ*msbB*Δ*eltA* OMV, anti-OMV mix, or anti-FliC (Creative Diagnostics) serum to achieve a final IgG1 concentration of 9 μg/ml. After 10–30 min incubation at room temperature, neonatal mice born to naive dams were infected with an infectious dose of approximately 8 × 10^4^ CFU/mouse. At 24 h post-infection, neonates were sacrificed and the colonization was determined as described above. To analyze the viability of ETEC in presence of anti-FliC sera, ETEC, and antisera were mixed as described above. After 30 min incubation at room temperature, samples were rigorously vortexed to dissolve potential aggregates and appropriate dilutions were plated for CFU counts.

### Preparation of blood, stool, and milk samples

Blood samples as well as fecal pellets were collected from immunized and sham-immunized adult mice throughout the immunization study and processed as previously described to monitor the induced immune response (Schild et al., 2008, 2009). Additionally, the stomach contents of infected mice were collected, pooled for each litter and the Igs were extracted by adding 300 μl of extraction buffer [PBS, 0.01% sodium azide, 5% fetal calf serum, one tablet complete EDTA-free protease inhibitor cocktail (Roche) per ml] per 100 mg of stomach content. The samples were disrupted by homogenization, centrifuged, and the clear supernatant (milk) was stored at −70°C.

### SDS-PAGE and immunoblot analysis

To analyze the protein content of OMVs, the standard sodium dodecyl sulfate-polyacrylamide gel electrophoresis (SDS-PAGE) procedure in combination with 15% gels and the Prestained Protein Marker Broad Range (New England BioLabs) as a molecular mass standard were used. Approximately 7 μg of each sample was loaded and either stained according to the procedure of Kang et al. or transferred to a nitrocellulose membrane (Amersham) for immunoblot analysis, which was performed as described previously (Kang et al., 2002; Roier et al., 2012).

### ELISA

Temporal immune responses of different Igs, half-maximum total Ig titers and mucosal immune responses to OMVs (5 μg/ml in PBS, pH 7.4) derived from *V. cholerae* and ETEC as well as the determination of the half-maximum total Ig titers to LPS (7 μg/ml in PBS, pH 7.4) of both organisms and the quantification of the IgG1 levels of purified proteins like His-CfaB (7 μg/ml in 25 mM Tris-HCl, pH 7.5) and His-FliC (7 μg/ml in PBS, pH 7.4) were carried out essentially as described previously, using horseradish peroxidase-conjugated goat anti-mouse antibodies (BD Biosciences) as secondary antibodies in combination with the TMB peroxidase substrate reagent set (BioLegend) (Roier et al., 2012; Leitner et al., 2013). Optical densities were monitored at 450 nm with a FLUOstar Omega microplate reader (BMG Labtech).

### Motility assay

The motility assay was performed with slight modifications as previously described (Leitner et al., 2013). To allow an adjustment to equal concentrations in the assay, the anti-IgG1 titer of each serum derived from mice immunized with OMVs and of the commercially available anti-FliC antibody (Creative Diagnostics) was determined via ELISA using the appropriate coating antigen (OMVs or His-FliC). Sera of the non-vaccinated control group were diluted by the lowest dilution factor needed for the immune serum to obtain 6 μg/ml anti-IgG1 titer in the same experiment. A GFP expressing ETEC strain was taken from a CFA plate supplemented with Cm, adjusted to an OD_600_ of 0.8 and mixed with heat-inactivated serum (56°C, 30 min) in a 2:1 ratio to achieve a final IgG1-titer of 6 μg/ml. After 10 min of incubation, 4 μl were placed on a slide under a coverslip and viewed with 60-x objective using a Nikon Eclipse TE300 microscope. The images were taken using the software NIS-Elements AR3.0.

### Statistical analysis

Data were analyzed using the Mann–Whitney U test or a Kruskal–Wallis test followed by *post-hoc* Dunn's multiple comparisons. Differences were considered significant at *P* < 0.05. For all statistical analyses, GraphPad Prism version 4.0a was used.

## Results

### Characterization of detoxified and enterotoxin negative mutants in *V. cholerae* and ETEC

**Figure 1:**
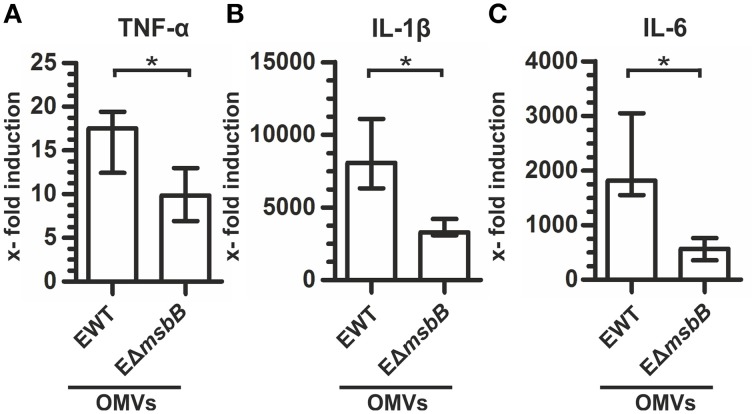
**EΔ***msbB*** OMVs are less endotoxic**. Shown is the induction of TNF-α **(A)**, IL-1β **(B)**, and IL-6 **(C)** in RAW macrophages after treatment with OMVs (1 μg/ml) derived from EWT or EΔ*msbB* compared to the non-stimulated control group and normalized to the housekeeping gene 36B4. Each data set represents the median from at least six independent experiments. The error bars indicate the interquartile range of each data set. Significant differences between the data sets are marked by asterisks (*P* < 0.05; Mann–Whitney U test).

We recently demonstrated that OMVs derived from *V. cholerae* lacking one functional secondary lipid A acyltransferase, MsbB (also referred to as LpxN), exhibit 50% less endotoxicity and retain their potential to induce a high-titer, protective immune response (Leitner et al., 2013). Encouraged by these results, we constructed a Δ*msbB* mutant strain in ETEC (EΔ*msbB*) to reduce the endotoxicity of the potential ETEC OMVs as vaccine candidates. To compare the endotoxicity of OMVs derived either from ETEC wild type (EWT) or the EΔ*msbB* mutant, the expression levels of the inflammatory markers TNF-α, IL-1ß, and IL-6 of OMV-stimulated RAW macrophages were analyzed (Figure 1). Non-stimulated RAW macrophages served as control. In concordance with previous results, the expression of all three cytokines was greatly induced by EWT OMVs, ranging from 18-fold for TNF-α to approximately 7500-fold for IL-1ß and almost 2000-fold for IL-6. In contrast, treatment with EΔ*msbB* OMVs induced a significantly weaker induction of an inflammatory response, which reached 10–50% of the level detected for EWT OMVs. This indicates a lower endotoxicity of the underacylated, detoxified LPS of EΔ*msbB* OMVs due to their reduced stimulation of TLR-4 (Somerville et al., 1996).

**Figure 2:**
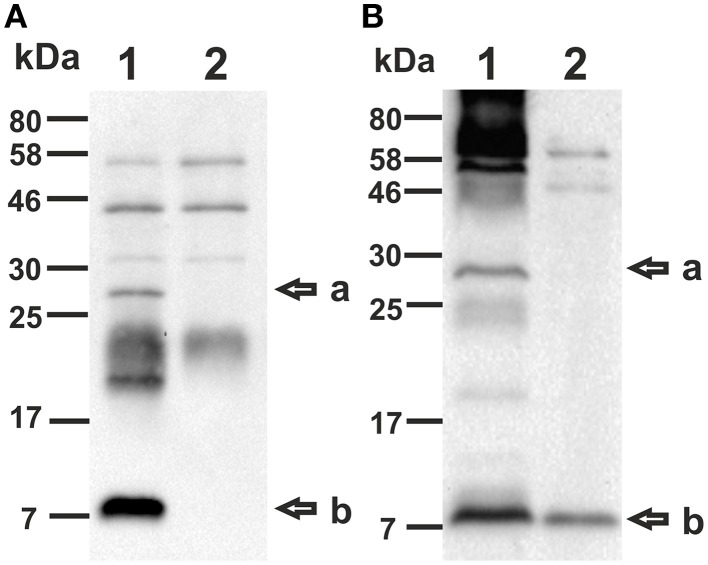
**Detection of CT and LT using immunoblot analysis**. Immunoblots were loaded with TCA precipitated supernatant of VWT (**A**, lane 1), VΔ*msbB*Δ*ctxAB* (**A**, lane 2), EWT (**B**, lane 1) or EΔ*msbB*Δ*eltA* (**B**, lane 2) and incubated with a LT cross-reactive anti-CT polyclonal antiserum (Sigma). The arrows to the right indicate the sizes of the A-subunit (a) as well as the B-subunit (b) of CT and LT. Lines to the left indicate the molecular masses of the protein standards in kDa. A corresponding Kang-stained gel (Kang et al., 2002) loaded with equal amounts as the immunoblot served as loading control and is presented in Figure S1.

Using the VΔ*msbB* and EΔ*msbB* mutants as a platform, we constructed OMV donor strains by additional deletion of both CT subunits in *V. cholerae* as well as the catalytically active A subunit of LT (LTA) in ETEC. The B subunit of LT was retained functional due to its reported adjuvant efficacy as well as it may provide cross-protection against *V. cholerae* as a result of the closely related AB-toxins of both pathogens (Clements and Finkelstein, 1978; Cheng et al., 1999). To visualize the absence of both CT subunits as well as LTA in the mutant strains, TCA precipitated culture supernatants were analyzed via immunoblot using the LT cross-reactive anti-CT polyclonal antisera (Sigma). This enabled the detection of both toxin subunits with the corresponding band at approximately 27 kDa for the A subunit and 11.6 kDa for the B subunit (Sixma et al., 1991) in the wild type samples of *V. cholerae* (VWT) and EWT (Figures 2A,B, lane 1). In contrast these bands were not detected in the samples derived from VΔ*msbB*Δ*ctxAB* and EΔ*msbB*Δ*eltA* mutant, confirming the absence of both CT subunits and the absence of LTA, respectively (Figures 2A,B, lane 2). The additional detection of bands are likely to be either cross-reacting bands appearing in wild type and respective mutant samples or might be CT-or LT-aggregates, in case of bands absent in the respective non-toxin mutant.

**Figure 3:**
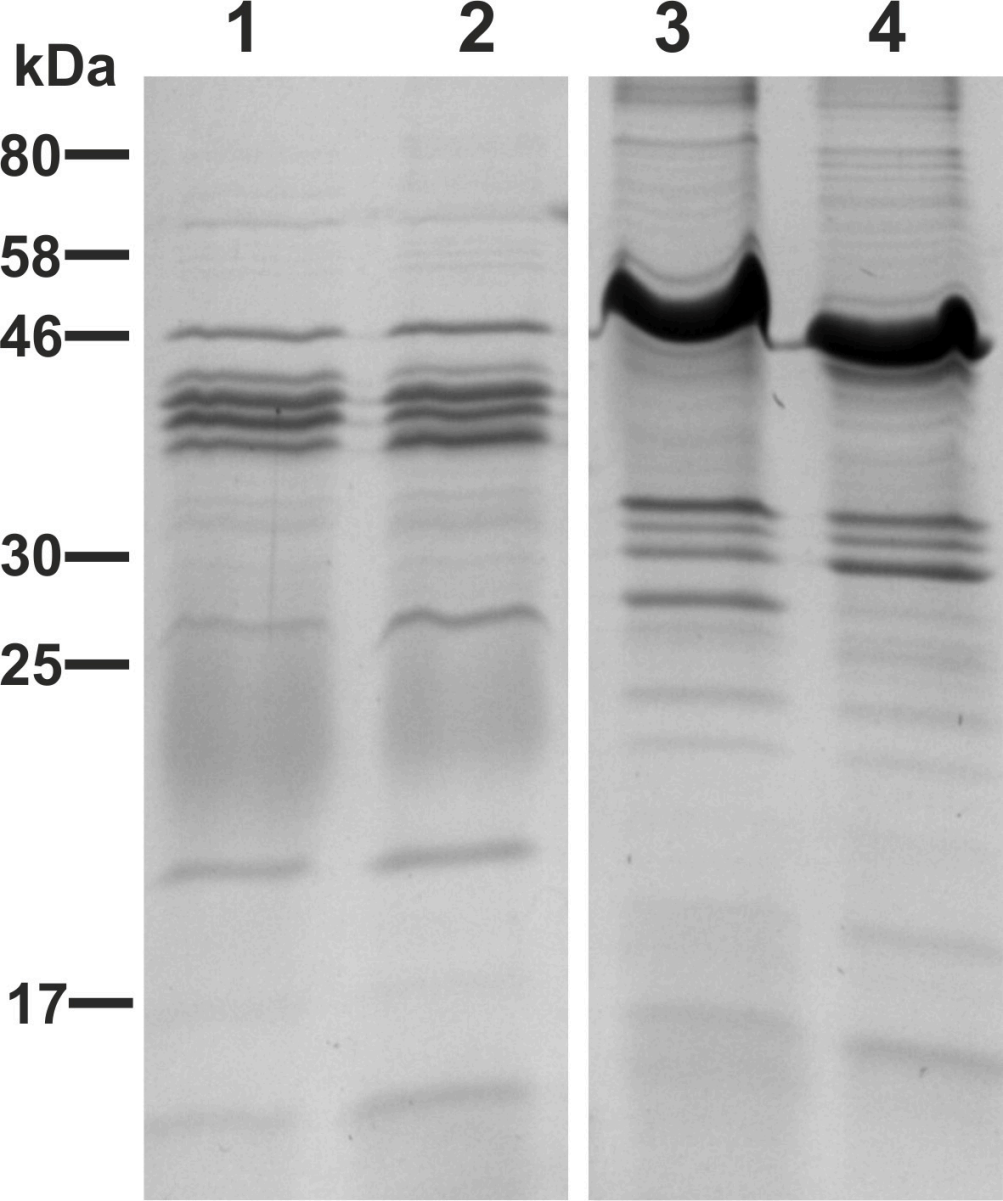
**OMV protein profiles of the OMV donor strains**. Depicted are the protein profiles of purified OMVs derived from VWT (lane 1), VΔ*msbB*Δ*ctxAB* (lane 2), EWT (lane 3) and EΔ*msbB*Δ*eltA* (lane 4). Samples (approximately 7 μg protein each) were separated by SDS-PAGE (15% gels) and protein bands were visualized according to Kang et al. (2002). Lines to the left indicate the molecular masses of the protein standard in kDa.

Furthermore, we analyzed the protein profile of the purified OMVs derived from VWT, VΔ*msbB*Δ*ctxAB*, EWT, and EΔ*msbB*Δ*eltA* by SDS-PAGE in combination with Kang‘s staining method (Figure 3). In concordance with previous data on *V. cholerae* OMVs, no obvious differences could be observed between OMVs from VWT and VΔ*msbB*Δ*ctxAB* (Figure 3, lanes 1 and 2) (Leitner et al., 2013). The protein profile of ETEC OMVs exhibits more high-molecular-weight bands, with the most prominent one at approximately 50 kDa (Figure 3, lanes 3 and 4). In comparison to the EWT OMV protein profile, the mutant lacks one band of approximately 27 kDa, which might be due to the *eltA* deletion. In general, the most abundant protein bands are present between 25 and 58 kDa in all OMV vaccine candidates (Figure 3, lanes 1–4). The LPS of EWT and EΔ*msbB*Δ*eltA* was also isolated and analyzed by silver staining, but no significant changes could be observed (data not shown).

### Immunization with OMVs of *V. cholerae* and ETEC induces a robust species-specific immune response

**Figure 4:**
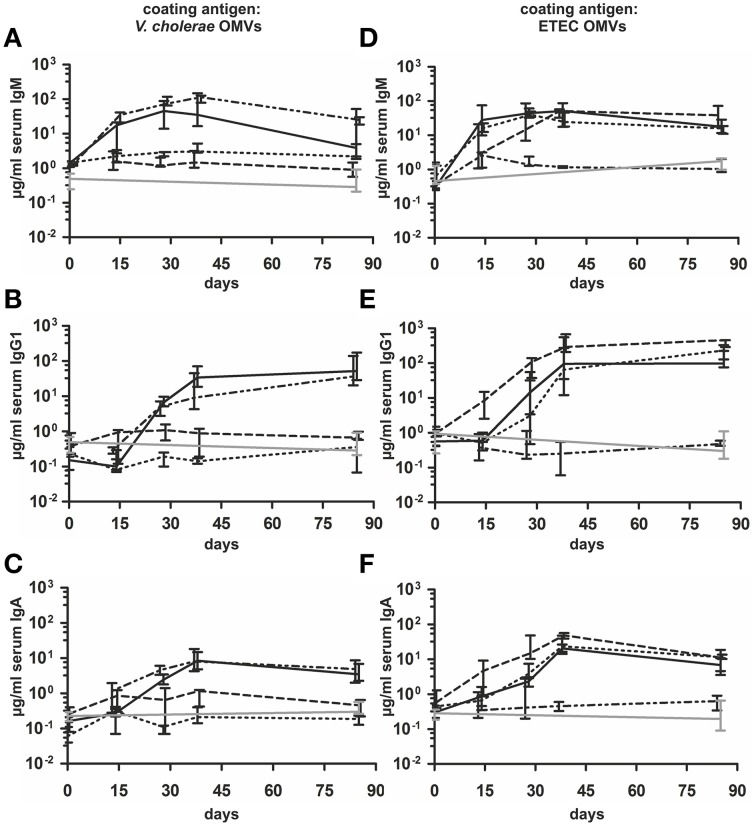
**Temporal immune responses to ***V. cholerae*** and ETEC OMVs**. Shown are the median titers over time of IgM **(A,D)**, IgG1 **(B,E)** and IgA **(C,F)** antibodies to OMVs derived from *V. cholerae* **(A–C)** and ETEC **(D–F)** in sera from mice intranasal immunized with VΔ*msbB*Δ*ctxAB* OMVs (dashed and dotted line), EWT OMVs (dashed line), EΔ*msbB*Δ*eltA* OMVs (dotted line), an OMV mix (black solid line) and from the non-vaccinated control group (gray solid line) (*n* = 8 for each group). The error bars indicate the interquartile range of each data set for each time point.

In order to investigate the immune response upon OMV immunization against both enteric pathogens, we intranasally immunized mice with OMVs derived from VΔ*msbB*Δ*ctxAB*, EWT, EΔ*msbB*Δ*eltA* strains or with a 1:1 mixture of VΔ*msbB*Δ*ctxAB* and EΔ*msbB*Δ*eltA* OMVs according to our standard immunization protocol (Schild et al., 2008). Mice sham-immunized with PBS served as non-vaccinated control group. To monitor the immune responses in sera of immunized and non-vaccinated mice by ELISA, OMVs derived from VWT or EWT were used as coating material. This allowed the detection of species and/or cross-species specific OMV antigen antibody responses. The temporal IgM, IgG1 and IgA responses in sera to OMVs derived from *V. cholerae* or ETEC are shown in Figure 4 for all immunization groups. The antibody titers of the non-vaccinated control group were determined for day 0 and day 86 and remained at very low levels for both time points. At day 0, the isotype-specific antibody titers to OMVs derived from *V. cholerae* or ETEC were relatively low and showed no significant differences between the tested immunization groups (*P* < 0.05; Kruskal–Wallis test and *post-hoc* Dunn's multiple comparisons). Mice immunized with VΔ*msbB*Δ*ctxAB* OMVs or the OMV mix induced an isotype-specific immune response against *V. cholerae* OMVs. In contrast, mice immunized with EWT, EΔ*msbB*Δ*eltA* OMVs or the OMV mix showed a pronounced increase of IgM, IgG1, and IgA titers against ETEC OMVs. Thus, immunization with *V. cholerae* or ETEC OMVs seems to induce a species-specific immune response. In general, the induction of an immune response was characterized by an Ig titer increase during the vaccination period with a peak at day 38 or day 78. Especially, IgG1 and IgA titers remained stable or even increased between day 38 and 78, which indicates the induction of a robust, long-lasting immune response. Mice immunized with EWT or EΔ*msbB*Δ*eltA* OMVs showed comparable induction patterns, indicating that the reduction of endotoxicity and the deletion of *eltA* did not affect the overall immunogenicity of ETEC OMVs.

**Figure 5:**
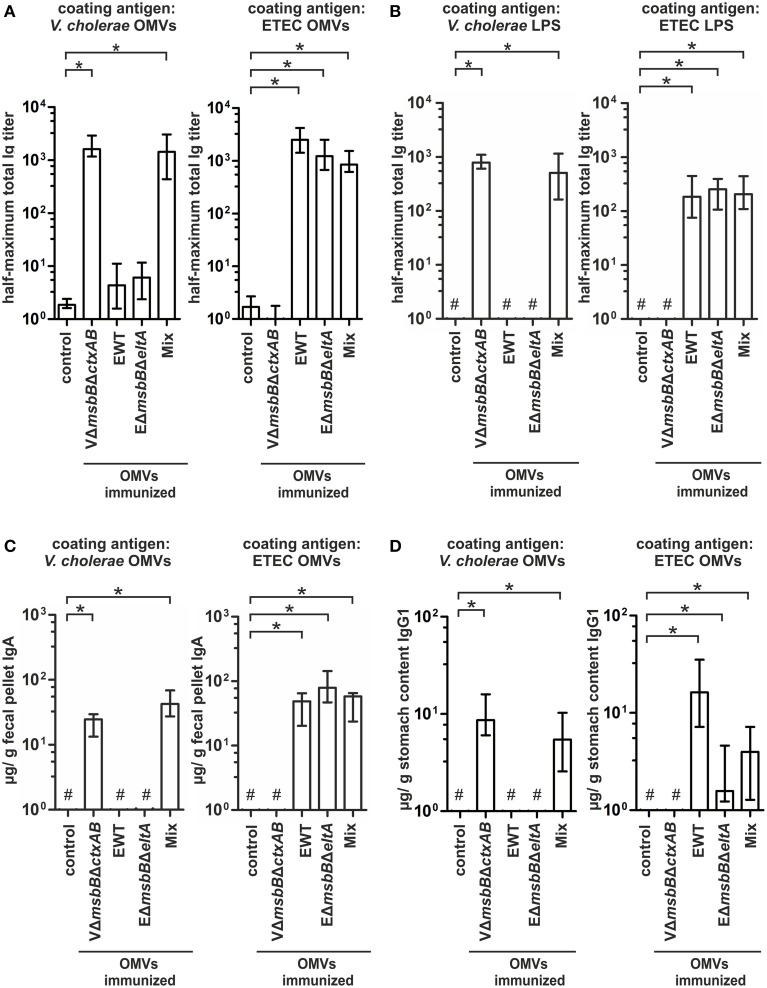
**Quantification of the immune response against ***V. cholerae*** and ETEC induced in OMV-immunized mice**. **(A,B)** Half-maximum total Ig titers to OMVs **(A)** derived from *V. cholerae* and ETEC as well as to purified *V. cholerae* or ETEC LPS **(B)** in sera collected at day 86 from mice intranasal immunized with VΔ*msbB*Δ*ctxAB* OMVs, EWT OMVs, EΔ*msbB*Δ*eltA* OMVs, and an OMV mix (*n* = 8 for each group). **(C)** Median IgA titers to OMVs derived from *V. cholerae* and ETEC extracted from fecal pellets collected at day 86 from the immunization groups (VΔ*msbB*Δ*ctxAB* OMVs, EWT OMVs, EΔ*msbB*Δ*eltA* OMVs, and the OMV mix) as well as from the non-vaccinated control group (*n* = 8 for each group). **(D)** IgG1 titers to OMVs derived from *V. cholerae* and ETEC in stomach contents collected from litters born from intranasal immunized female mice of the immunization groups (VΔ*msbB*Δ*ctxAB* OMVs, EWT OMVs, EΔ*msbB*Δ*eltA* OMVs, and the OMV mix) as well as the non-vaccinated control group during the challenge period (day 67–78). The error bars indicate the interquartile range of each data set and the hash key that the result of the data set was below the limit of detection. Significant differences between the data sets are marked by asterisks (*P* < 0.05; Kruskal–Wallis test and *post-hoc* Dunn's multiple comparisons).

**Figure 6:**
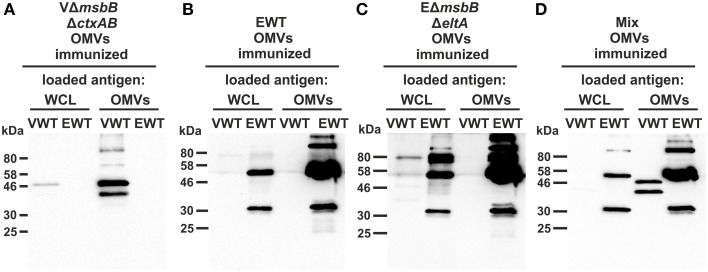
**Characterization of the antibody response against surface proteins of ***V. cholerae*** and ETEC in sera from OMV-immunized mice**. Representative immunoblots were loaded with *V. cholerae* (VWT) as well as ETEC (EWT) whole cell lysates (WCL) and OMVs (approximately 7 μg protein each) as indicated above each blot and incubated with sera collected at day 86 from mice immunized with VΔ*msbB*Δ*ctxAB* OMVs **(A)**, EWT OMVs **(B)**, EΔ*msbB*Δ*eltA* OMVs **(C)**, and an OMV mix **(D)**. Lines to the left indicate the molecular masses of the protein standards in kDa. A corresponding Kang-stained gel (Kang et al., 2002) loaded with equal amounts as the immunoblot served as loading control and is presented in Figure S2.

To further characterize the induced humoral immune response in sera, the half-maximum total Ig titers against *V. cholerae* or ETEC OMVs as well as against purified *V. cholerae* or ETEC LPS were quantified (Figures 5A,B). In addition, the mucosal immune response against *V. cholerae* and ETEC was analyzed by monitoring the secretory IgA titers in fecal pellets of immunized mice as well as the IgG1 titers in the stomach content of neonates, displaying the Ig transfer from the immunized dams to the offspring via breast milk (Figures 5C,D). As previously noted (Schild et al., 2009), the ELISA results of the stomach contents exhibited more variation than those for fecal pellets. In general, a similar induction pattern for the sera samples, fecal pellets, and milk samples was observed for each immunization group. In comparison to the non-vaccinated control group the V*msbB*Δ*ctxAB* OMV immunization group showed significant higher titers against *V. cholerae*, but not against ETEC. In contrast, the groups immunized with EWT or EΔ*msbB*Δ*eltA* OMVs exhibited a significant induction of Ig titers against ETEC, but not against *V. cholerae*, if compared to the non-vaccinated control group. In concordance with the temporal immune response only the OMV mix group induced a robust immune response against both pathogens. Notably, this group exhibited a similar immune response for all three isotypes in comparison to the respective immunization group receiving only a single OMV type (i.e., VΔ*msbB*Δ*ctxAB* OMVs, EΔ*msbB*Δ*eltA* OMVs, or EWT OMVs) and the respective coating material. These results suggest no adverse effects by mixing *V. cholerae* and ETEC OMVs regarding their induction of a species-specific immune response. Finally, immunoblot analysis using whole cell lysates (WCL) and OMVs derived from *V. cholerae* and ETEC as antigens were used to analyze the specificity of the antibody response against proteins present in the respective samples. These immunoblots were incubated with sera collected at day 86 from one representative mouse of each immunization group as well as from the non-vaccinated control group (Figure 6). No bands were detected on immunoblots using sera of the non-vaccinated control group mice (data not shown). Immunoblots incubated with sera from groups immunized with *V. cholerae* OMVs allowed detection of bands in the protein profile of *V. cholerae*, whereas no bands were detected against ETEC. Immunoblots incubated with sera from the EWT or EΔ*msbB*Δ*eltA* OMV immunization group revealed various detectable bands in the protein profile of ETEC, but only low-intensity bands for *V. cholerae* samples. This is concordant with the ELISA results and suggests no significant induction of a cross-species-specific antibody response by using just OMVs from one species. Immunoblots incubated with sera of the OMV mix group showed intensive bands against *V. cholerae* and ETEC samples, highlighting the induction of an immune response against both pathogens. In addition, the OMV mix and the VΔ*msbB*Δ*ctxAB* OMV immunization group showed comparable band patterns against *V. cholerae* samples. The same could be observed for the OMV mix, the EWT as well as EΔ*msbB*Δ*eltA* OMV immunization groups against ETEC samples. Thus, the OMV mix group induced a similar specificity of the immune response on the protein level, if compared to the individual species-specific immune response upon immunization with either *V. cholerae* or ETEC OMVs. In general, immunoblots loaded with WCL and OMV samples derived from the same strain revealed similar patterns, but the intensity of the detected bands was always stronger when OMVs were loaded as antigen, indicating that the immune response was mainly directed against surface-associated structures highly abundant in OMVs.

### Immunization with an OMV mix induces a protective immune response

**Figure 7:**
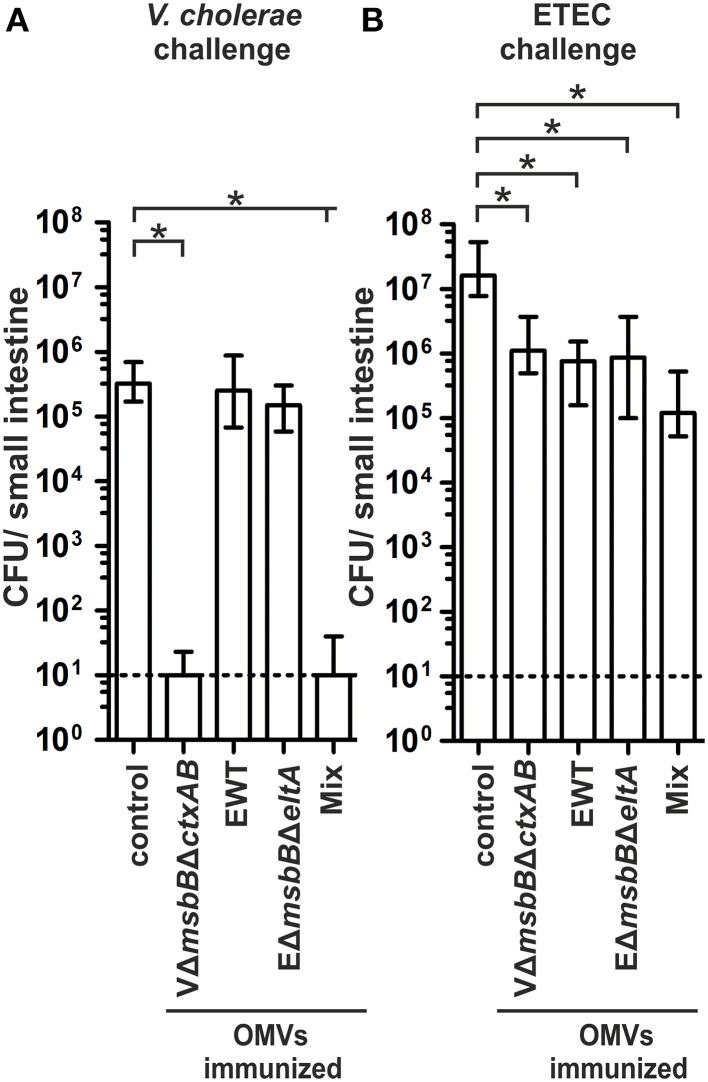
**Challenge of neonates born to OMV immunized mice with ***V. cholerae*** and ETEC**. Shown are the numbers of recovered CFU/small intestine for neonates born to mice immunized with VΔ*msbB*Δ*ctxAB* OMVs, EWT OMVs, EΔ*msbB*Δ*eltA* OMVs, and an OMV mix as well as the non-vaccinated control group, which were challenged with either *V. cholerae* **(A)** or ETEC **(B)** (*n* = 11 for each group). When no bacteria were recovered, the number of CFU was set to the limit of detection of 10 CFU/small intestine (dotted line). The error bars indicate the interquartile range of each data set. Significant differences between the data sets are indicated by asterisks (*P* < 0.05; Kruskal–Wallis test and *post-hoc* Dunn's multiple comparisons).

We used the infant mouse model to investigate whether the induced humoral immune response also results in protection against a *V. cholerae* or ETEC infection (Schild et al., 2008, 2009; Bishop et al., 2010; Leitner et al., 2013). This indirect protection assay was used, since adult mice are only successfully colonized by these human pathogens after pretreatment with antibiotics to decrease the bacterial gut flora. Therefore, the offspring of immunized dams were challenged orally and the level of protection was measured by the degree of colonization in the small intestine. To allow a continuous transfer of Igs from the immunized dams to the suckling offspring, the infected neonatal mice were given back to their respective dams for the challenge period. In order to gain challenge data for both enteric pathogens, the offspring of each immunization group were divided into two subgroups and challenged orally with *V. cholerae* or ETEC using approximately 9 × 10^4^ CFU/mouse. These infection doses are at least 100-fold above the ID_50_ and have been used for previous challenge experiments (Goldhar et al., 1986; Schild et al., 2009; Roy et al., 2011; Leitner et al., 2013). In the case of a challenge with *V. cholerae*, neonatal mice of the non-vaccinated control group as well as of the EWT and EΔ*msbB*Δ*eltA* OMV immunization groups were colonized at comparable levels of around 10^5^–10^6^ CFU/small intestine (Figure 7A). In contrast, neonatal mice from the VΔ*msbB*Δ*ctxAB* OMV and the OMV mix group were completely protected from colonization with *V. cholerae* or showed at least a 1000-fold reduction in their colonization level (*P* < 0.05; Kruskal–Wallis test and *post-hoc* Dunn's multiple comparisons). Challenging neonatal mice from dams of the non-vaccinated control group with an ETEC strain resulted in a stable colonization with a median colonization level of around 10^7^ CFU/small intestine (Figure 7B), whereas neonatal mice from dams immunized with either VΔ*msbB*Δ*ctxAB*, EWT or EΔ*msbB*Δ*eltA* OMVs showed a significant 10-fold reduction in the colonization level (*P* < 0.05; Kruskal–Wallis test and *post-hoc* Dunn's multiple comparisons). The highest reduction in the colonization rate upon ETEC challenge was observed in the OMV mix group. Here, a 100-fold reduction in viable CFU counts/small intestine compared to the non-vaccinated control group was observed.

### *V. cholerae* OMVs can be used for heterologous expression of ETEC surface antigens

Based on the induction of a species-specific immune response as described above, we hypothesized that OMVs derived from one species and decorated with heterologously expressed antigens from the other species could induce a cross-protective immune response. Using VΔ*msbB*Δ*ctxAB* OMVs as antigen delivery vehicle, we genetically engineered the strain to express either the CFA/I fimbriae or the hybrid flagellin FlaA-FliC (H) on these OMVs. For the latter case, *flaA* was deleted on top of the VΔ*msbB*Δ*ctxAB* mutant to avoid influence of the intrinsic major flagellin FlaA. Furthermore, the hybrid flagellin was used to allow an efficient transport of the flagellin to the outer membrane. Presence of these surface antigens in OMV preparations was verified by immunoblot analysis using the respective OMVs in combination with an anti-CfaB or an anti-FliC antibody (Figure S3). The amount of the heterologous expressed proteins was approximately 3–5% of the protein composition of the analyzed OMVs.

**Figure 8:**
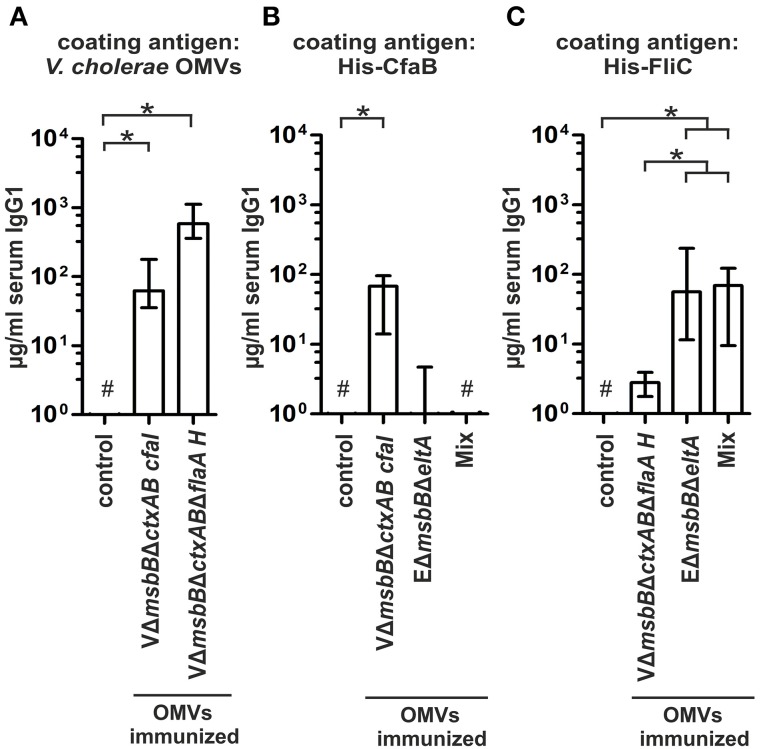
**Quantification of the immune response of ***V. cholerae*** OMVs expressing ETEC surface antigens. (A)** Depicted are the IgG1 titers in sera collected at day 86 from mice intranasal immunized with VΔ*msbB*Δ*ctxAB* CFA/I OMVs, VΔ*msbB*Δ*ctxAB*Δ*flaA* hybrid FlaA-FliC OMVs and from the non-vaccinated control group to *V. cholerae OMVs* (*n*≥8 for each group). The error bars indicate the interquartile range of each data set and the hash key that the result of the data set was below the limit of detection. Significant differences between the data sets are indicated by asterisks (*P* < 0.05; Kruskal–Wallis test and *post-hoc* Dunn's multiple comparisons). **(B,C)** Shown are the IgG1 titers to purified His-CfaB **(B)** and His-FliC **(C)** in sera collected at day 86 from mice intranasal immunized with VΔ*msbB*Δ*ctxAB* CFA/I OMVs, VΔ*msbB*Δ*ctxAB*Δ*flaA* hybrid FlaA-FliC OMVs, EΔ*msbB*Δ*eltA* OMVs, an OMV mix, or the non-vaccinated control group (*n*≥8 for each group). The error bars indicate the interquartile range of each data set and the hash key that the result of the data set was below the limit of detection. Significant differences between the data sets are indicated by asterisks (*P* < 0.05; Kruskal–Wallis test and *post-hoc* Dunn's multiple comparisons).

In order to elucidate the protective potential of the CFA/I or of the hybrid flagellin decorated *V. cholerae* OMVs, female mice were intranasally immunized with these OMVs according to our standard immunization protocol. Serum samples, fecal pellets as well as milk samples were collected throughout the immunization study to monitor the humoral and the mucosal immune response. No obvious differences could be detected in the temporal, total, or the secretory immune response against *V. cholerae* or ETEC compared to the VΔ*msbB*Δ*ctxAB* OMV immunization group, representing the undecorated OMV type (data not shown). Both immunization groups exhibited significantly higher IgG1 titers against *V. cholerae* OMVs compared to the non-vaccinated control group (*P* < 0.05; Kruskal–Wallis test and *post-hoc* Dunn's multiple comparisons) (Figure 8A). In contrast, the antibody response against ETEC OMVs was below the limit of detection. Thus, the heterologous expression of ETEC antigens on *V. cholerae* OMVs has no adverse effect on the induction of a high-titer immune response against the OMV donor species, but fails to induce a overall detectable immune response against ETEC OMVs.

To investigate the induction of an immune response against the heterologous antigens, we determined the IgG1 titers against purified His-CfaB and His-FliC in sera of immunized and non-vaccinated mice (Figures 8B,C). Besides the respective groups immunized with the CFA/I or the hybrid flagellin decorated *V. cholerae* OMVs, the EΔ*msbB*Δ*eltA* OMV immunization group and the OMV mix group were also included in this analysis. The group immunized with CFA/I decorated *V. cholerae* OMVs showed a significant induction of the IgG1 titer against His-CfaB compared to the non-vaccinated control group (Figure 8B) (*P* < 0.05; Kruskal–Wallis test and *post-hoc* Dunn's multiple comparisons). Additionally, a weak anti-CfaB response was observed in some mice immunized with EΔ*msbB*Δ*eltA* OMVs, whereas none was detected in the OMV mix group. In contrast, all tested immunization groups (VΔ*msbB*Δ*ctxAB*Δ*flaA* hybrid FlaA-FliC OMV-, EΔ*msbB*Δ*eltA* OMV immunization group and the OMV mix group) showed a significant IgG1 response against purified His-FliC compared to the non-vaccinated control group (Figure 8C) (*P* < 0.05; Kruskal–Wallis test and *post-hoc* Dunn's multiple comparisons). The anti-FliC titers in the EΔ*msbB*Δ*eltA* OMV or OMV mix immunization groups were significantly higher compared to the VΔ*msbB*Δ*ctxAB*Δ*flaA* hybrid FlaA-FliC OMV immunization group (*P* < 0.05; Kruskal–Wallis test and *post-hoc* Dunn's multiple comparisons). In summary, a detectable immune response against each of the heterologously expressed antigens could be induced. In contrast to CfaB, which does not seem to serve as an antigen in ETEC OMVs, FliC represented a highly immunogenic structure of the OMV species. We also analyzed the immune response against proteins present in the OMVs and the antibody specificity raised against the LPS structure by immunoblot and ELISA, respectively. Both assays revealed no differences compared to the results of the VΔ*msbB*Δ*ctxAB* OMV immunization group (data not shown).

**Figure 9:**
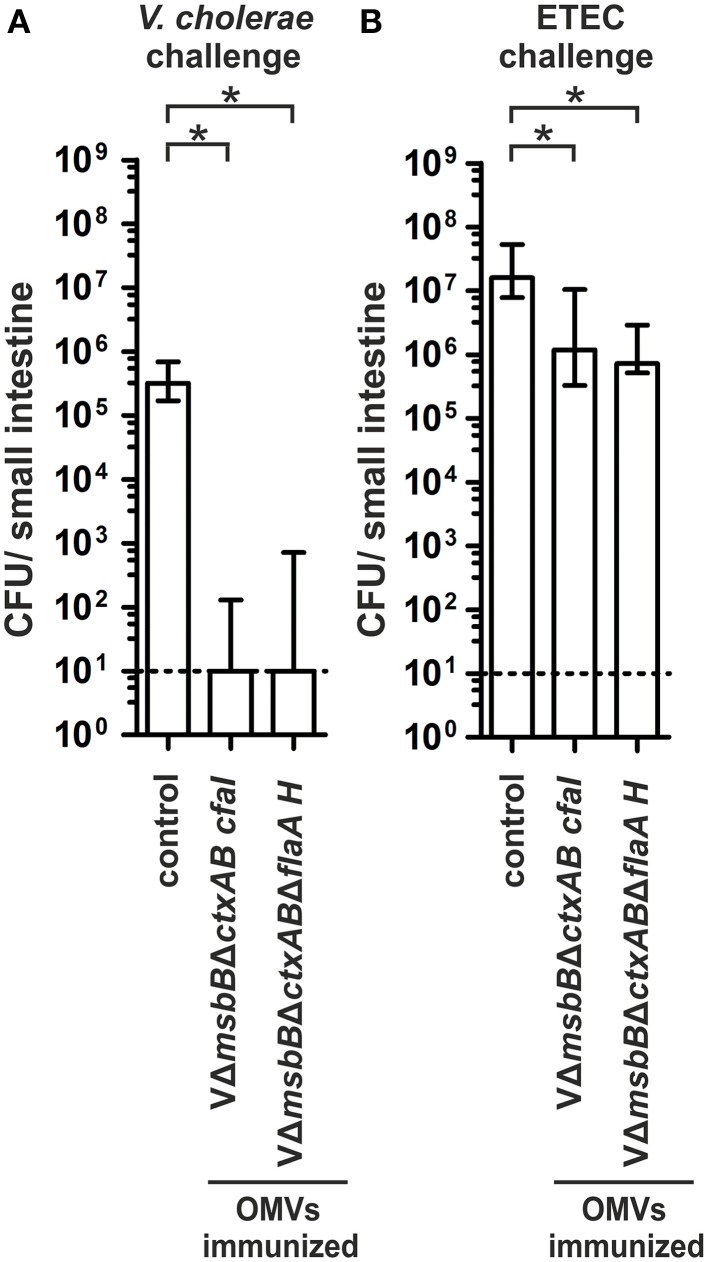
**Challenge of neonates born to mice immunized with ***V. cholerae*** OMVs expressing ETEC antigens**. Shown are the numbers of recovered CFU/small intestine for neonates born to mice immunized with VΔ*msbB*Δ*ctxAB* CFA/I OMVs, VΔ*msbB*Δ*ctxAB*Δ*flaA* hybrid FlaA-FliC OMVs as well as the non-vaccinated control group mice, which were challenged with either *V. cholerae* **(A)** or ETEC **(B)** (*n*≥11 for each group). When no bacteria were recovered, the number of CFU was set to the limit of detection of 10 CFU/small intestine (dotted line). The error bars indicate the interquartile range for each data set. Significant differences between the data sets are indicated by asterisks (*P* < 0.05; Kruskal–Wallis test and *post-hoc* Dunn's multiple comparisons).

In order to investigate whether the induced immune responses confer protection against a *V. cholerae* or ETEC infection, we orally challenged the offspring of the immunized dams according to the infant mouse model described above (Figures 9A,B). All non-vaccinated control mice were stably colonized with median colonization rates of 10^5^ or 10^7^ CFU/small intestine for *V. cholerae* and ETEC, respectively. In contrast, both immunization groups challenged with *V. cholerae* showed a significant reduction of their colonization rate or were completely protected from colonization compared to the non-vaccinated control group (Figure 9A) (*P* < 0.05; Kruskal–Wallis test and *post-hoc* Dunn's multiple comparisons). Challenging neonatal mice of dams immunized with decorated *V. cholerae* OMVs with ETEC resulted in a significant 10-fold reduction in the colonization level, which has already been observed in the VΔ*msbB*Δ*ctxAB* immunization group (Figure 9B) (*P* < 0.05; Kruskal–Wallis test and *post-hoc* Dunn's multiple comparisons). Thus, neither the immunization of CFA/I nor the hybrid flagellin decorated *V. cholerae* OMVs resulted in a higher reduction in the colonization rate upon ETEC challenge.

### Inhibition of motility *in vitro* correlates with the anti-flic titer in sera of OMV immunized mice and contributes to protection *in vivo*

**Figure 10:**
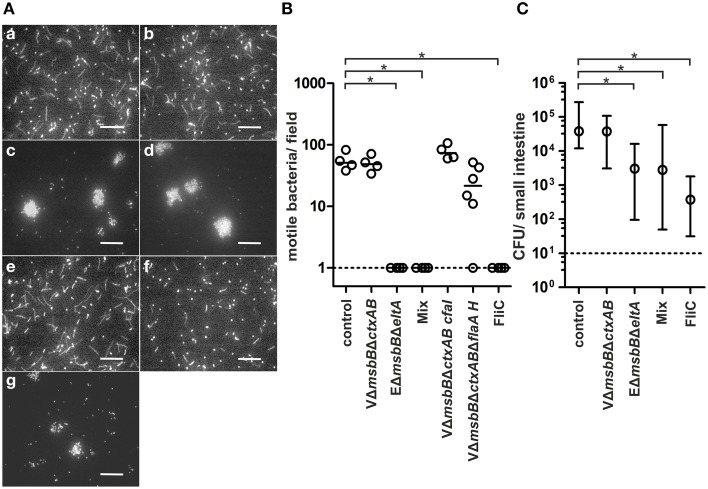
**Inhibition of motility ***in vitro*** correlates with the presence of anti-FliC antibodies and contributes to protection ***in vivo*****. **(A)** Representative images using ETEC mixed with non-vaccinated control sera (a), sera of VΔ*msbB*Δ*ctxAB* OMV immunized mice (b), sera of EΔ*msbB*Δ*eltA* OMV immunized mice (c), sera of the OMV mix group (d), sera of VΔ*msbB*Δ*ctxAB* CFA/I OMV immunized mice (e), sera of VΔ*msbB*Δ*ctxAB*Δ*flaA* hybrid FlaA-FliC OMV immunized mice (f), and anti-FliC antibodies (g). The bacterial motility was visualized by fluorescence microscopy. Motile bacteria appeared as swirls and lines and non-motile bacteria as dots. Bars, 50 μm. **(B)** Quantification of the bacterial motility of ETEC cells in the presence of non-vaccinated control sera, sera of VΔ*msbB*Δ*ctxAB* OMV immunized mice, sera of EΔ*msbB*Δ*eltA* OMV immunized mice, sera of the OMV mix group, sera of VΔ*msbB*Δ*ctxAB* CFA/I OMV immunized mice, sera of VΔ*msbB*Δ*ctxAB*Δ*flaA* hybrid FlaA-FliC OMV immunized mice and anti-FliC antibodies. Each symbol represents an independent experiment, and the horizontal bar indicates the median of each data set. When no bacteria were visible, the number was set to the limit of detection of 1 bacterium/field (dotted line). Significant differences between the data sets are indicated by asterisks (*P* < 0.05; Kruskal–Wallis test and *post-hoc* Dunn's multiple comparisons). **(C)** Passive immunization of neonates born to naïve dams using ETEC WT mixed with sera of the non-vaccinated control group, sera of VΔ*msbB*Δ*ctxAB* OMV immunized mice, sera of EΔ*msbB*Δ*eltA* OMV immunized mice, sera of the OMV mix group or anti-FliC antibodies (*n*≥6 for each group). The error bars indicate the interquartile range of each data set. Significant differences between the data sets are indicated by asterisks (*P* < 0.05; Kruskal–Wallis test and *post-hoc* Dunn's multiple comparisons).

We recently demonstrated that protection of neonatal mice from *V. cholerae* infection occurs through the inhibition of bacterial motility by O antigen antibodies from OMV immunized dams, which bind to the LPS present in the outer membrane sheath of the single polar flagellum of *V. cholerae* (Leitner et al., 2013). Since motility is crucial for the efficient adhesion of ETEC to intestinal cells (Haiko and Westerlund-Wikström, 2013) and based on the high anti-FliC levels in ETEC OMV immunized mice, we hypothesized that blockage of ETECs' flagella could also play an important role in protection against ETEC infection. To test our hypothesis, we performed an established *in vitro* motility assay in which pooled sera of OMV immunized or non-vaccinated mice as well as flagellum specific antibodies were mixed 1:2 ratio with a bacterial suspension of a GFP expressing ETEC strain and examined by fluorescence microscopy (Figure 10A) (Leitner et al., 2013). Motile bacteria appeared as swirls or lines, whereas non-motile bacteria appeared as bright dots. We also quantified the number of motile bacteria per field in each image (Figure 10B). Sera from non-vaccinated control mice served as control and did not result in any inhibition of motility. Only sera from EΔ*msbB*Δ*eltA* OMV immunized mice or the OMV mix group, which contain FliC antibodies, as well as the commercially available antisera directed against FliC resulted in complete blockage of motility. Neither serum from VΔ*msbB*Δ*ctxAB*, nor VΔ*msbB*Δ*ctxAB* CFA/I immunized mice reduced the motility of ETEC. Interestingly, we only observed a slight reduction of the motility, when serum of mice immunized with VΔ*msbB*Δ*ctxAB*Δ*flaA* hybrid FlaA-FliC OMVs, containing FliC antibodies, was used for the *in vitro* motility assay. This might be explained by the 10-fold lower anti-FliC titer of the VΔ*msbB*Δ*ctxAB*Δ*flaA* hybrid FlaA-FliC OMV immunization group compared to the EΔ*msbB*Δ*eltA* OMV immunized group or the OMV mix group (Figure 8C). Thus, we excluded the VΔ*msbB*Δ*ctxAB*Δ*flaA* hybrid FlaA-FliC OMV immunization group for further analysis. In summary, our results demonstrate that the inhibition of motility *in vitro* correlates with the presence of an anti-FliC titer.

To determine whether anti-FliC antibodies might also play a role in the protection against ETEC *in vivo*, we performed a passive immunization study using the infant mouse model (Figure 10C). Naive infant mice were subdivided into five groups and challenged with a defined dose of EWT mixed with one of the five antisera used in the motility assay, separately. The level of protection was determined by the degree of colonization in the small intestine after 24 h. Mice passively immunized with either the sera from the non-vaccinated control group or the VΔ*msbB*Δ*ctxAB* OMV immunized group showed a high colonization of 10^5^ CFU/small intestine, whereas mice passively immunized with sera from the EΔ*msbB*Δ*eltA* group or the OMV mix group showed a significantly reduction in their colonization (*P* < 0.05; Kruskal–Wallis test and *post-hoc* Dunn's multiple comparisons). The highest reduction in the colonization rate upon ETEC challenge was observed when mice were passively immunized with the commercially available anti-FliC antibody. Here, we could demonstrate a 100-fold reduction in viable CFU counts/small intestine compared to the non-vaccinated control group (*P* < 0.05; Kruskal–Wallis test and *post-hoc* Dunn's multiple comparisons). To exclude the possibility that ETEC CFUs are already altered in the inoculation mix upon presence of anti-FliC sera, viability of ETEC was determined by plating for CFU counts after 30 min of incubation. No significant reduction in CFU for anti-FliC sera treated samples compared to the PBS control could be observed (Figure S4). Thus, the observed effects cannot be explained by reduced viability upon contact with this antisera. In summary, the inhibition of motility based on anti-FliC antibodies contributes to the observed protection against ETEC.

## Discussion

Over the last years OMVs have been intensively studied as experimental vaccine candidates against various Gram-negative pathogens. Encouraged by the promising results, we herein describe the first combined vaccine approach based on OMVs derived from different OMV donor species namely *V. cholerae* and ETEC. Naturally, these native OMVs are composed of large amounts of fully endotoxic LPS with a hexa-acylated lipid A moiety, which is a very potent activator of TRL-4 (Raetz and Whitfield, 2002). Although this renders the LPS an effective adjuvant, it mainly contributes to vaccine reactogenicity as an excessive activation of the inflammatory response may result in septic shock. Therefore, the reduction of the endotoxicity is an essential step toward a safe application of an OMV vaccine candidate in humans. The LPS content can be reduced either by detergent extraction, which is applied for the production of the classical *N. meningitides* OMV vaccines (Fredriksen et al., 1991; Oster et al., 2005) or by genetically detoxification of the LPS (Van Der Ley et al., 2001; Fisseha et al., 2005; van der Ley and van den Dobbelsteen, 2011). Recently, we investigated genetically detoxified OMVs of *V. cholerae* and demonstrated that the non-stimulatory LPS produced by the *msbB* mutant strain retain sufficient antigenicity to serve as protective target for vaccination. Based on these results, we genetically modified the lipid A structure of ETEC by deleting the secondary lipid A acyltransferase, MsbB, and analyzed the endotoxic activity of the resulting strain. In concordance with VΔ*msbB* OMVs (Leitner et al., 2013), OMVs derived from an EΔ*msbB* strain reduced the induction of an inflammatory response by 50% compared to EWT OMVs without affecting the induction of a protective immune response.

In this study, we focused on mucosal immunization strategies due to a simple administration and a robust induction of secretory Ig titers. Since ETEC OMVs are tightly associated with active LT (Horstman and Kuehn, 2000, 2002; Kesty et al., 2004), which can cause severe adverse effects including facial palsy, the safety of the intranasal delivery of the OMV vaccine candidate was brought into question for human applications (Mutsch et al., 2004; Lewis et al., 2009). Although a recent study did not report any side effects using 5 μg LT for intranasally immunization of mice, an OMV vaccine candidate with active LT is unlikely to be approved (Roy et al., 2011). Thus, we pursued our immunization studies with an ETEC OMV vaccine candidate, lacking the enzymatically active subunit *eltA* of LT on top of the *msbB* deletion. The remaining B subunit in the ETEC OMV vaccine candidate serves as an adjuvant as well as it may provide cross-protection against *V. cholerae* due to the closely related AB-toxins of these two pathogens (Clements and Finkelstein, 1978). In the case of *V. cholerae*, it already has been demonstrated that CT is not required as an adjuvant for successful *V. cholerae* OMV immunization, since OMVs from a CT mutant are as immunogenic and protective as VWT OMVs (Bishop et al., 2010). Based on a recent report, which suggest that the CT of *V. cholerae* could be associated with the OMVs, we used a CT deficient and detoxified *V. cholerae* OMV donor strain in this study (Chatterjee and Chaudhuri, 2011).

To test the immunogenic and protective properties of *V. cholerae* as well as ETEC OMVs, we performed a nasopharyngeal immunization study, wherein female BALB/c mice were immunized either with OMVs derived solely from one species or an OMV mix, and analyzed the protection resulting from this acquired immunity. The immunization with VΔ*msbB*Δ*ctxAB* OMVs, EWT OMVs, or EΔ*msbB*Δ*eltA* OMVs did not induce a cross-protective antibody response against ETEC or *V. cholerae*, respectively. In contrast, the use of an OMV mix resulted in a robust immune response with protective efficacy against both pathogens. Remarkably, this group showed a similar induction pattern of the humoral as well as the secretory immune response in comparison to the respective immunization group receiving only a single OMV type (i.e., VΔ*msbB*Δ*ctxAB* OMVs, EΔ*msbB*Δ*eltA* OMVs or EWT OMVs) and the respective coating material. Furthermore, this group exhibited a protective immune response against *V. cholerae*, which was comparable with the VΔ*msbB*Δ*ctxAB* OMV immunization group as well as the highest protective response against ETEC observed in this study. Taken together, these results demonstrate that the OMV mix has the ability to induce a protective high-titer species-specific immune response against both pathogens, although just half of the amount of *V. cholerae* or ETEC OMVs has been used for the immunization compared to the immunization group receiving only a single OMV type.

Surprisingly, the use of VΔ*msbB*Δ*ctxAB* OMVs alone already induced a so far uncharacterized protective mechanism against ETEC colonization. In general, such a cross-protection was observed previously and thought to be due to cross-reactive antibodies targeting the conserved B-subunit of the respective toxins (Peltola et al., 1991). However, we used a non-toxigenic *V. cholerae* mutant in this study. Thus, a CTB dependent mechanism explaining the 10-fold reduction of the ETEC colonization level can be excluded. While the EWT OMV or EΔ*msbB*Δ*eltA* OMV immunization groups stimulate the induction of species-specific antibodies, this cannot be the case for the VΔ*msbB*Δ*ctxAB* OMV immunization group, since we have not observed a detectable antibody response against ETEC. Thus, the mechanism of this protective immune response is unlikely based on the induction of anti-ETEC OMV antibodies, but might be explained by antibody-independent mechanisms. This hypothesis can be further encouraged by the passive protection study, where the anti-VΔ*msbB*Δ*ctxAB* OMV sera did not affect the colonization efficiency of ETEC in the small intestine.

In an alternative approach, *V. cholerae* OMVs decorated with the ETEC-specific antigens CFA/I or FliC were tested for the induction of a cross-protective immune response. Both antigens have been suggested as vaccine determinants and several studies have demonstrated their protective effects (Ahrén and Svennerholm, 1982; Tacket et al., 1988; Freedman et al., 1998; Roy et al., 2009a; Svennerholm, 2011). In general, we observed a similar induction of the humoral and the secretory immune response against *V. cholerae* compared to the VΔ*msbB*Δ*ctxAB* OMV immunization group, but the titers against ETEC OMVs remained below the limit of detection. Additionally, a detectable immune response against the heterologously expressed antigens could be observed and revealed FliC being a highly immunogenic structure compared to CFA/I in ETEC OMVs. However, these approaches have not resulted in an improved protection against an ETEC challenge compared to a simple *V. cholerae* and ETEC OMV mixture. Since this approach did not yield in an increased protective immune response, we did not comprehensively analyze the exact localization (surface-accessible or luminal) of the heterologously expressed antigens, which might affect immunogenicity. Furthermore, it could be speculated that a larger amount of the heterologously expressed antigens would have resulted in higher antibody titers against the presented antigen and might have induced a cross-species specific immune response with an improved protection against an ETEC challenge. Furthermore, it is questionable if every foreign antigen is suitable for the incorporation into OMVs as the immunogenicity of the delivery vehicle in comparison to the presented antigen might be crucial for the induction of a sufficient immune response against both. Thus, the development of a high-titer immune response against the used surface antigens was probably hampered by a higher immunogenicity of *V. cholerae* OMVs compared to the heterologously expressed antigens (Dhungyel et al., 2014).

Recently, we identified the O antigen being the dominant protective antigen of the OMV based cholera vaccine candidate and revealed a protection mechanism based on the inhibition of motility (Leitner et al., 2013). Since motility is crucial for the efficient adhesion of ETEC to intestinal cells (Haiko and Westerlund-Wikström, 2013) and based on the high anti-FliC levels in ETEC OMV immunized mice, we speculated that the blockage of ETEC flagella could also play an important role in protection against ETEC infection. In concordance with this hypothesis, the sera of mice immunized with EΔ*msbB*Δ*eltA* OMVs or the OMV mix blocked motility, while sera of the non-vaccinated control group, the VΔ*msbB*Δ*ctxAB* OMV or the VΔ*msbB*Δ*ctxAB* CFA/I OMV immunization groups showed no effect. If sera of mice immunized with VΔ*msbB*Δ*ctxAB*Δ*flaA* hybrid flagellin OMVs were used an intermediate phenotype was observed. Thus, we hypothesized that a certain titer of anti-FliC antibodies targeting the major flagellin, FliC, is crucial for the inhibition of motility. By using a defined anti-FliC antiserum, we were able to confirm this hypothesis. Furthermore, we could demonstrate that the observed inhibition of motility correlates with the anti-FliC antibody titer as those immunization groups, which efficiently blocked motility showed a higher immune response against purified His-FliC compared to the VΔ*msbB*Δ*ctxAB*Δ*flaA* hybrid flagellin OMV immunization group. The crucial role of anti-FliC antibodies in protection against ETEC infection is highlighted by the passive immunization study. Sera containing a sufficient amount of anti-FliC antibodies (e.g., EΔ*msbB*Δ*eltA* OMVs, the OMV mix or the anti-FliC) are capable of reducing the colonization of ETEC. Consequently, the inhibition of motility via anti-FliC antibodies represents a relevant protection mechanism of an OMV-based ETEC vaccine candidate. In the case of the OMV mix group, which comprises anti-ETEC as well as anti-*V. cholerae* OMV antibodies, we have observed a 100-fold reduction of the colonization level in our primary immunization study. Thus, we hypothesize that the 100-fold reduction of the ETEC colonization is due to a combination of an antibody-dependent (ETEC OMVs) as well as an antibody-independent (*V. cholerae* OMVs) immune response.

Although our results indicate that the major flagellin represents a protective antigen of an OMV based ETEC vaccine, flagellar organelles have previously been excluded in the development of ETEC vaccines due to their high variation in H serotypes and the assumption that only variant instead of highly conserved FliC regions are exposed. Nonetheless, these highly conserved FliC regions are potent stimuli of the immune response and mice immunized with a full-length flagellin of one serotype have been significantly protected from a subsequent challenge with an ETEC strain expressing another flagellin serotype (Roy et al., 2009a,b). Thus, our findings support these results and open a future direction in the ETEC vaccine development including a flagellin, which would engender an immune response directed against the highly conserved regions of the antigen. Therefore, ETEC OMVs can be seen as an attractive vaccine alternative, since they stimulate the immune system with a combination of multiple antigens including FliC, resulting in a protective antibody response. This idea is further emphasized by recent immunoproteomic studies, which demonstrated that the immune response to ETEC is quite complex and involves the recognition of multiple proteins (Roy et al., 2010). Thus, the inclusion of various antigenic components should be the focus of an efficient broadly protective ETEC vaccine. Hence, OMVs represent ideal vaccine candidates, which can be easily isolated with any modifications from different donor species and combined in various OMV mixtures. Based on the results of this study, the use of such a combined OMV mixture is highly recommended as the OMV mix group represents the only immunization group inducing a protective immune response against both pathogens. It could be speculated that the protective immune response might be even more pronounced by adjusting the ratio of the respective OMVs for immunization based on their immunogenicity. Furthermore, this idea could be extended to develop a broadly protective OMV vaccine candidate against several Gram-negative pathogens by combining their OMVs. Thus, the results of this study not only provide first steps toward an inexpensive broad-spectrum OMV vaccine candidate against *V. cholerae* and ETEC, but also against other Gram-negative pathogens of interest.

### Conflict of interest statement

The authors declare that the research was conducted in the absence of any commercial or financial relationships that could be construed as a potential conflict of interest.
