# Supplementary material for: A combined vaccine approach against Vibrio cholerae and ETEC based on outer membrane vesicles
Source: Front Microbiol. 2015 Aug 11;6:823. doi: 10.3389/fmicb.2015.00823 (PMC4531250; doi:10.3389/fmicb.2015.00823)
Supplement: Supplementary file 1 [file Image1.PDF]

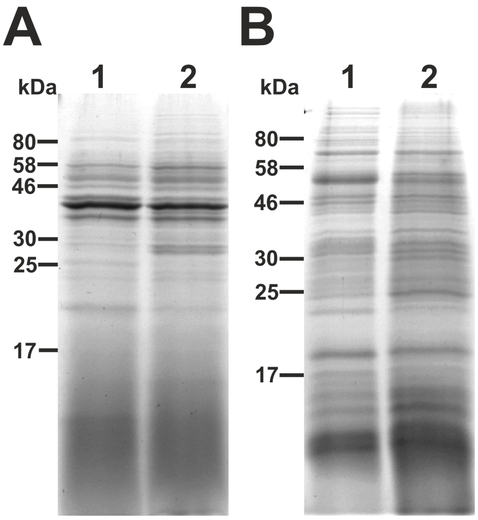

Figure S1: **Protein profile of the TCA precipitated supernatants.** Depicted are the protein profiles of the TCA precipitated supernatants of VWT (panel A, lane 1), VΔ*msbB*Δ*ctxAB* (panel A, lane 2), EWT (panel B, lane 1) or EΔ*msbB*Δ*eltA* (panel B, lane 2). Samples were separated by SDS-PAGE (15% gels) and protein bands were visualized according to Kang et al. (Kang et al., 2002). Lines to the left indicate the molecular masses of the protein standard in kDa. The corresponding immunoblot is presented in figure 2.
