# Supplementary material for: A combined vaccine approach against Vibrio cholerae and ETEC based on outer membrane vesicles
Source: Front Microbiol. 2015 Aug 11;6:823. doi: 10.3389/fmicb.2015.00823 (PMC4531250; doi:10.3389/fmicb.2015.00823)
Supplement: Supplementary file 2 [file Image2.PDF]

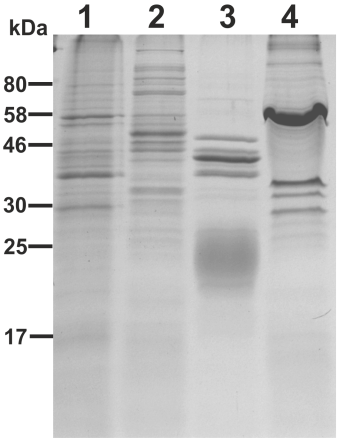

Figure S2: **Protein profile of the whole cell lysates and the OMVs derived from *V. cholerae* and ETEC.** Shown are the protein profiles of VWT (whole cell lysate, lane 1; OMVs, lane 3) and EWT (whole cell lysate, lane 2; OMVs, lane 4). Samples were separated by SDS-PAGE (15% gels) and protein bands were visualized according to Kang et al. (Kang et al., 2002). Lines to the left indicate the molecular masses of the protein standard in kDa. The corresponding immunoblot is presented in figure 6.
