# Supplementary material for: A combined vaccine approach against Vibrio cholerae and ETEC based on outer membrane vesicles
Source: Front Microbiol. 2015 Aug 11;6:823. doi: 10.3389/fmicb.2015.00823 (PMC4531250; doi:10.3389/fmicb.2015.00823)
Supplement: Supplementary file 3 [file Image3.PDF]

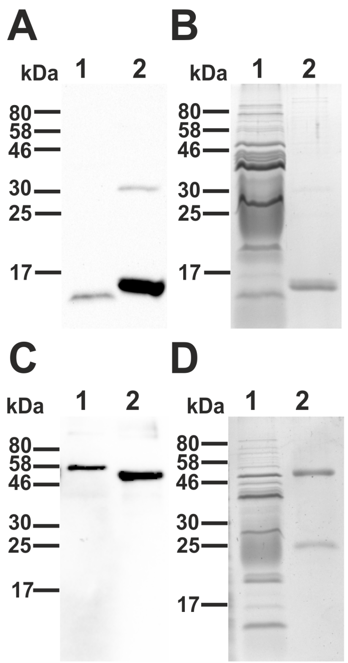

Figure S3: **Quantification of the heterologous ETEC antigens present on *V. cholerae* OMVs.** Depicted are the immunoblots for the detection of CfaB **(A)** or FliC **(C)** as well as the protein profiles after Kang staining **(B and D)** of VΔ*msbB*Δ*ctxAB* CFA/I OMVs and purified HIS-CfaB **(B)** as well as VΔ*msbB*Δ*ctxAB*Δ*flaA* hybrid FlaA-FliC OMVs and purified HIS-FliC **(D)**. Samples (7 µg protein equivalent of OMVs and 1 µg of the purified protein) were separated by SDS-PAGE (15% gels) and protein bands were visualized according to Kang et al. (Kang et al., 2002). Lines to the left indicate the molecular masses of the protein standard in kDa. The intensities of the respective detected protein bands were determined using the Image Lab software (Bio-Rad Laboratories) in order to calculate the amount present in the respective OMV sample using the purified protein (1 µg) as reference. Based on the analysis the respective band in the OMV samples exhibit 1/5 (CfaB) or 1/3 (FliC) of the intensity of the purified protein reference, respectively. This correlates with approx. 200 ng (CfaB) and 330 ng (FliC) heterolougously expressed antigen present in 7 µg of the respective OMV sample, which corresponds to approximately 3–5% heterologously expressed antigen with respect to the overall protein amount of the analyzed OMVs.
