# Supplementary material for: A combined vaccine approach against Vibrio cholerae and ETEC based on outer membrane vesicles
Source: Front Microbiol. 2015 Aug 11;6:823. doi: 10.3389/fmicb.2015.00823 (PMC4531250; doi:10.3389/fmicb.2015.00823)
Supplement: Supplementary file 4 [file Image4.PDF]

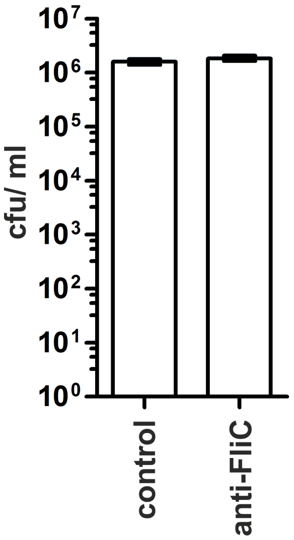


Figure S4: **ETEC viability is not altered by treatment with anti-FliC sera.** Depicted are the median CFU/ml after incubation with PBS or anti-FliC sera. The error bars indicate the interquartile range of each data set (n=6).
